# Biomechanical modelling of the facet joints: a review of methods and validation processes in finite element analysis

**DOI:** 10.1007/s10237-020-01403-7

**Published:** 2020-11-22

**Authors:** Marlène Mengoni

**Affiliations:** Institute of Medical and Biological Engineering, University of Leeds, Leeds, UK

**Keywords:** Facet joints, Validation, Variability, Finite element, Reproducibility

## Abstract

There is an increased interest in studying the biomechanics of the facet joints. For *in silico* studies, it is therefore important to understand the level of reliability of models for outputs of interest related to the facet joints. In this work, a systematic review of finite element models of multi-level spinal section with facet joints output of interest was performed. The review focused on the methodology used to model the facet joints and its associated validation. From the 110 papers analysed, 18 presented some validation of the facet joints outputs. Validation was done by comparing outputs to literature data, either computational or experimental values; with the major drawback that, when comparing to computational values, the baseline data was rarely validated. Analysis of the modelling methodology showed that there seems to be a compromise made between accuracy of the geometry and nonlinearity of the cartilage behaviour in compression. Most models either used a soft contact representation of the cartilage layer at the joint or included a cartilage layer which was linear elastic. Most concerning, soft contact models usually did not contain much information on the pressure-overclosure law. This review shows that to increase the reliability of *in silico* model of the spine for facet joints outputs, more needs to be done regarding the description of the methods used to model the facet joints, and the validation for specific outputs of interest needs to be more thorough, with recommendation to systematically share input and output data of validation studies.

## Introduction

There is an increased interest in analysis of the facet joints in biomechanical studies of the spine. The facet joints (zygapophysial joints) constitute with the intervertebral disc the three joints complex of the functional spinal unit (motion segment). They are synovial joints located posterior to the vertebrae and the intervertebral disc and contribute to the motion and stability of the spine. Osteoarthritis of the facet joints is thought to be a widespread cause of back pain (Gellhorn et al. 2013), in part because of its high prevalence and early development, with facet joints degeneration associated with a radiological narrowing of the joint space (Pathria et al. 1987). Studies have shown association between intervertebral disc degeneration and facet osteoarthritis, even though the latter can exist without the former (Jaumard et al. 2011b; Gellhorn et al. 2013). Moreover, some disc treatments leading to adjacent disc disease are thought to exacerbate facet osteoarthritis (O’Leary et al. 2018).

Finite element analysis of spine biomechanics can be useful to assess different scenario for a range of spinal disorders or associated surgical interventions (e.g. among many others Rohlmann et al. 2006b; Bashkuev et al. 2018 or Ottardi et al. 2016; Calvo-Echenique et al. 2018, respectively). It can complement *in vitro* or *in vivo* experiments with scenario testing and inclusion of a wider variation in the anatomy and tissue degeneration.

Key requirements for using *in silico* models in clinical or preclinical contexts are the assessment of their credibility defined from a clear understanding of their applicability (Morrison et al. 2019) and known validation processes (Jones and Wilcox 2008; Henninger et al. 2010). Validation of *in silico* model is the process of making sure that the right equations are solved, and the correct parameters are used for a given scenario. A model is never “valid” for all possible scenarios and applications; a validation process is linked to a specific question of interest (Viceconti et al. 2020).

Finite element models of the multi-level spinal unit include different levels of complexity, either in the material behaviour or in the type of tissues included in models. Most finite element models of the human spine are validated against range of motion (e.g. Ayturk and Puttlitz 2011; Azari et al. 2018; Barthelemy et al. 2016; Holzapfel and Stadler 2006; Khoddam-Khorasani et al. 2018; Noailly et al. 2012; Rohlmann et al. 2006a; Schmidt et al. 2013), facet joint forces (e.g. Ayturk and Puttlitz 2011; Azari et al. 2018; Barthelemy et al. 2016) or intervertebral disc pressure (e.g. Ayturk and Puttlitz 2011; Azari et al. 2018; Khoddam-Khorasani et al. 2018; Liu et al. 2018; Rohlmann et al. 2006a); with the majority of work comparing their outcome to experimental or computational data available in the literature. This provides a validation process which gives confidence that models can predict outcomes within a range of values for given outputs of interest.

The aim of this review was to assess the methods used in modelling the facet joints in finite element models of multi-level spinal units, and the validation processes used for the facet joints biomechanics in such models. To avoid assessing the same model used for different clinical scenarios, only original models (with model development presented for the first time) and their validation studies were included in this review. With its focus on methods and validation, this work is complementary to recent reviews on the role of each spinal component in load transmission (Ghezelbash et al. 2020), on the structure–function relationship of the facet joints (O’Leary et al. 2018), or on finite element analysis of the cervical spine biomechanics (Suarez-Escobar and Rendon-Velez 2018; Kim et al. 2018).

## Methods

**Fig. 1:**
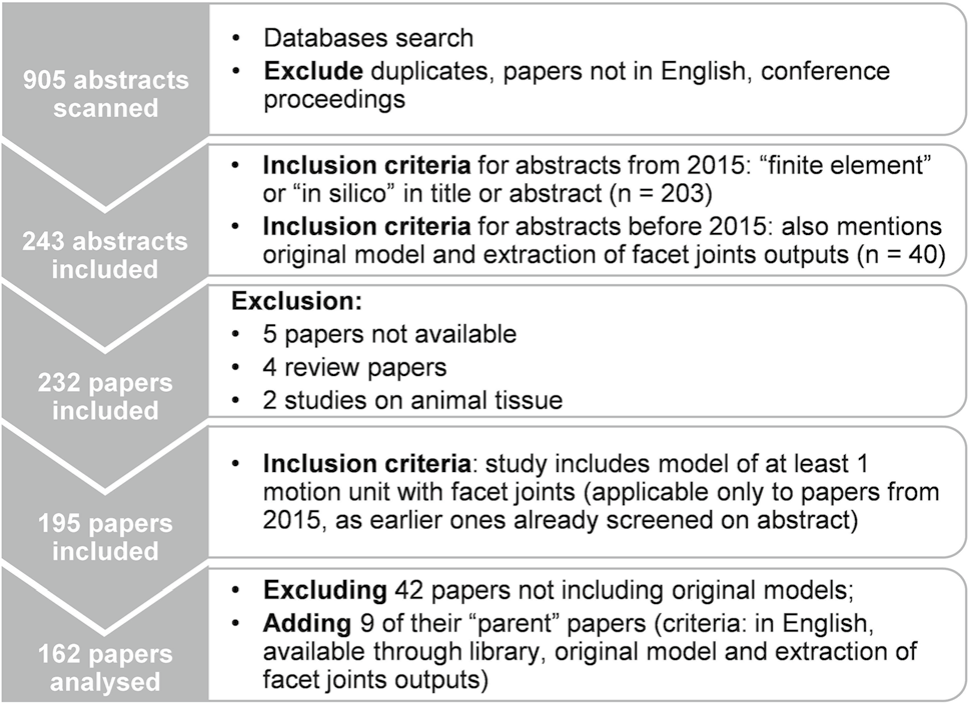
Inclusion and exclusion criteria

Three databases (PubMed, Web of Science and Scopus) were searched for papers up to August 2020 with keywords [“finite element” or computational] AND [“facet joint” or zygapophysial] (Fig. 1). Papers not written in English, duplicates, and conference proceedings were excluded. Due to a previous literature search supporting a computational validation study for ovine facet joints in 2015 (Mengoni et al. 2016), different criteria were used to include papers based on their titles and abstracts up to 2014 or from 2015: the more recent were screened for containing keywords “finite element” or “*in silico*”, while the older ones were screened to also contain explicit information about facet joints outputs and model development (by opposition to using an existing model). From the remaining papers, 11 were excluded because they were not available through the University library $(n = 5)$, were reviews $(n = 4)$ or were animal models $(n = 2)$. Full papers were screened to include only those which were studies of at least one functional unit with facet joints $(n = 195)$ and presented original models $(n = 153)$. From papers excluded because of the latter criteria, nine new “parent” papers were included, after screening for explicitly containing information about facet joints outputs and new model development.

**Fig. 2:**
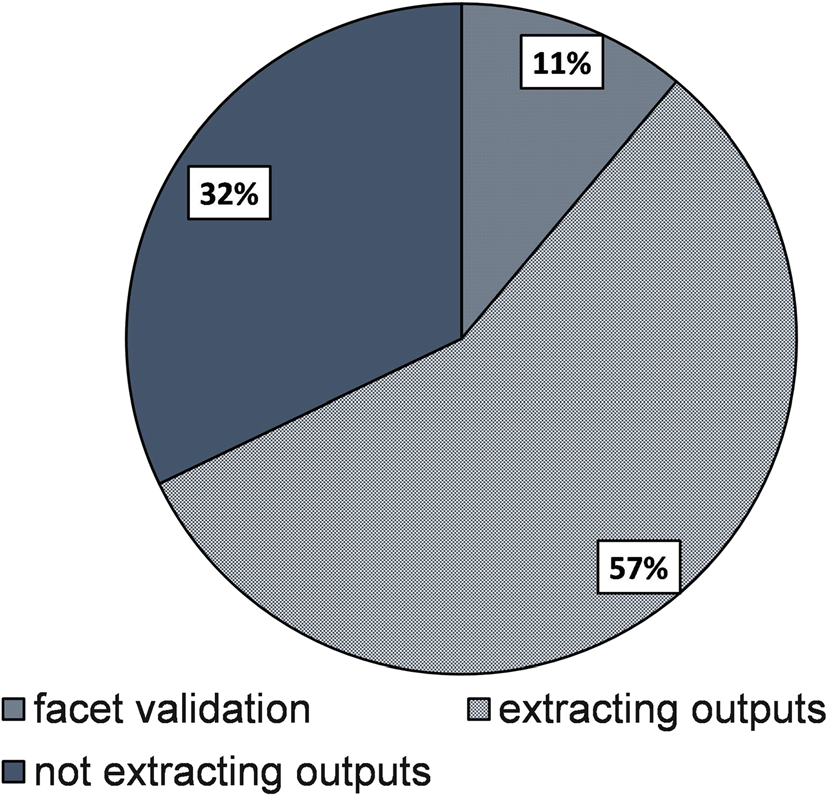
Analysed papers contained studies which did not extract outputs of interest for facet joints (32%) or studies with validation of facet joints biomechanics (11%)

Of the 162 analysed papers (Fig. 2), 52 did not contain outputs of interest related to the facet joints (all from 2015 given the differential in inclusion criteria) and are not reported here. In summary, from an initial set of 905 abstracts, 110 studies were analysed in this review (47 published before 2015), 18 of which contained some validation method on facet joints biomechanical behaviour: just under one in six original models using facet outputs had some validation for the facet joints.

Key aspects of the modelling methodology for facet joints were extracted: geometry representation, contact model, and, when relevant, cartilage material model. Attention was paid to the validation processes for facet joints outputs of interest.

## Methodologies for facet joints models

### Facet joint representation

Of studies reporting relevant information, just over half (*n* = 50/95, Table 1) did not include the cartilage explicitly but represented its function through contact pairs and a soft-contact pressure-overclosure model. The studies which explicitly included cartilage (*n* = 45/95, Table 2) usually modelled its behaviour as a linear elastic material (*n* = 37/45). Quite a few studies (*n* = 15/110, Table 3) did not include sufficient information to know how the facet joint was represented (i.e. studies that may mention some information about friction but not if cartilage was represented explicitly or through a soft contact model, or that mention the presence of cartilage without information on material model used).

When cartilage was included explicitly, it was always reconstructed from the bone anatomy, and from reported observation of facet joint space and/or cartilage thickness in facet joints.

### Facet joint contact model

Whether or not the cartilage is included explicitly, a contact model is required to represent the interaction between opposite facet surfaces.

**Table 1:** Contact models for studies which do not explicitly include a cartilage layer at the facet joints (empty cells refer to a lack of relevant information)

References	Friction model	Pressure-overclosure information
Azari et al. (2018)	Frictionless	Initial joint space of 0.6 mm
Campbell et al. (2016)	Frictionless	Initial joint space as per CT; linear formulation
Cao et al. (2020)		Initial joint space of 0.5 mm
Chen et al. (2002)		Initial joint space of 1 mm
Chen et al. (2009)	Friction coefficient of 0.1	Initial joint space of 0.5 mm
Du et al. (2016b)	Frictionless	Initial joint space of 0.5 mm
Galbusera et al. (2008)	Frictionless	
Goel et al. (1988)	Frictionless	Initial joint space of 0.45 mm
Guo et al. (2007)	Frictionless	
Guo and Li (2020)	Frictionless	Initial joint space of 0.5 mm
Khoddam-Khorasani et al. (2018)		Max gap of 1.25 mm
Kim et al. (2012)		Exponential formulation
Kim et al. (2017)	Frictionless	Exponential formulation
Kong et al. (1998)		Initial joint space of 1.25 mm, contact initiated at gap of 0.75 mm, exponential formulation, pressure at zero gap of 35 GPa
Liu et al. (2011)	Friction coefficient of 0.1	Initial joint space of 0.5 mm
Liu et al. (2018)	Frictionless	Max gap of 1.5 mm
Liu et al. (2020)	Frictionless	Initial joint space of 0.5 mm
Mustafy et al. (2014)	Frictionless	
Naserkhaki et al. (2016)	Frictionless	Max gap of 2 mm
Niemeyer et al. (2012)	Frictionless	Initial joint space of 0.01 mm to 0.4 mm (uniform probability distribution), contact initiated for overclosure of 0.01 to 0.3 mm (uniform probability distribution), exponential formulation, pressure at zero gap of 170 MPa
Nikkhoo et al. (2019)	Frictionless	Initial joint space of 0.3 mm, exponential formulation
Nikkhoo et al. (2020)	Frictionless	Initial joint space of 0.5 mm, contact initiated at gap of 0.5 mm, exponential formulation, pressure at zero gap of 120 MPa
Rohlmann et al. (2006b)		Initial joint space of 0.5 mm, gap/pressure curve with a pressure at zero gap of 12 GPa
Rundell et al. (2011)	Frictionless	
Sharma et al. (1995)		Initial joint space of 0.6 mm, contact initiated at gap of 0.4 mm, gap/pressure curve with a pressure at zero gap of 12 GPa
Shen et al. (2019)	Frictionless	
Shirazi-Adl et al. (1986)	Frictionless	
Shirazi-Adl and Drouin (1987)	Frictionless	Initial joint space of 1 mm, max overclosure 0.5 mm
Shirazi-Adl (1994)	Frictionless	Contact initiated at overclosure of 1.25 mm, compression moduli of 75 and 150 MPa
Sterba et al. (2019)	Frictionless	
Teo et al. (2003)	Frictionless	Normal penalty increasing linearly from 0 to 12 GPa at overclosure of 0.2 mm
Tsouknidas et al. (2015)	Friction coefficient of 0.1	Initial joint space of 0.5 mm
Wang et al. (1997)	Frictionless	Initial joint space of 0.4 mm, pressure at zero gap 1.6 MPa
Wang et al. (2020)	Friction coefficient of 0.1	
Xu et al. (2017)	Frictionless	Initial joint space of 0.5 mm
Zhu et al. (2017)	Frictionless	Initial joint space 0.5 mm

When cartilage was not included, the pressure-overclosure model in the soft contact formulation represents both the direct contact behaviour and the compliance of the cartilage in compression. It was rarely described with sufficient details (Table 1): most studies included information about initial joint space (initial bony gap with no physical cartilage present) or maximum overclosure values (max gap) but usually did not provide much information about the pressure-overclosure relationship. Fourteen studies (out of 49) did not provide any other information than using a “soft contact” or “non-linear contact” formulation (Aroeira et al. 2018; Bermel et al. 2018; Campbell and Petrella 2016; Charles et al. 2013; Cheung et al. 2003; Deng et al. 2017; Goto et al. 2002; Kim et al. 1991; Lo et al. 2019; Pitzen et al. 2002; Song et al. 2014, 2018; Teo and Ng 2001; Zeng et al. 2017). Only six of the 50 models with cartilage behaviour modelled as soft contact reported all required information on the pressure-overclosure law, of which only four also reported friction behaviour.

**Table 2:** Material model and parameters, and friction models of studies explicitly including a cartilage layer at the facets (empty cells refer to a lack of relevant information)

References	Friction model	Cartilage material model
Bashkuev et al. (2018, 2020)	Frictionless	Linear elastic: *E* variable, $\nu$ = 0.3
Cai et al. (2020)	Friction coefficient of 0.01	Linear elastic: $E=10\,\text {MPa}$, $\nu$ = 0.4
Calvo-Echenique et al. (2018); Kang et al. (2015, 2017); Kim et al. (2013); Schmidt et al. (2012)	Frictionless	Linear elastic: $E = 35\,\text {MPa}$, $\nu$ = 0.4
Ezquerro et al. (2011)	Frictionless	Linear elastic
Guo et al. (2019)	Frictionless	Linear elastic: $E = 10\,\text {kPa}$
Huang et al. (2018); Li et al. (2017b, 2018); Mo et al. (2017); Rong et al. (2017); Wu et al. (2017)	Frictionless	Linear elastic: $E = 10.4\,\text {MPa}$, $\nu$ = 0.4
John et al. (2018)		Linear elastic: $E = 10\,\text {MPa}$, $\nu$ = 0.3
Kang et al. (2010)		Linear elastic: $E = 0.5\,\text {MPa}$, $\nu$ = 0.45
Lee et al. (2011, 2016); Li et al. (2017c); Sun et al. (2020)		Linear elastic: $E = 10.4\,\text {MPa}$, $\nu$ = 0.4
Li et al. (2017a)	Friction coefficient of 0.07	Linear elastic: $E = 10.4\,\text {MPa}$, $\nu$ = 0.4
Li et al. (2019)	Frictionless	Linear elastic: $E = 10\,\text {MPa}$, $\nu$ = 0.4
Mesfar and Moglo (2013)		Linear elastic: $E = 10\,\text {MPa}$, $\nu$ = 0.45
Mills and Sarigul-Klijn (2019)		Linear elastic: $E = 35\,\text {MPa}$, $\nu$ = 0.4
Moumene and Geisler (2007); Park et al. (2013b)		Linear elastic: $E = 11\,\text {MPa}$, $\nu$ = 0.4
Ottardi et al. (2016)		Linear elastic: $E = 23.8\,\text {MPa}$, $\nu$ = 0.4
Panzer and Cronin (2009)	Squeeze-film-bearing model + frictionless	Linear elastic: $E = 10\,\text {MPa}$, $\nu$ = 0.4
Park et al. (2013a)	Frictionless	Linear elastic: $E = 11\,\text {MPa}$, $\nu$ = 0.4
Tang and Meng (2011)	Frictionless	Linear elastic: $E = 3500\,\text {MPa}$, $\nu$ = 0.25
Wang et al. (2013)	Frictionless	Linear elastic: $E = 75\,\text {MPa}$, $\nu$ = 0.4
Wang et al. (2016a)	Frictionless	Linear elastic
Wang et al. (2016b)	Friction coefficient of 0.01	Linear elastic: $E = 23.8\,\text {MPa}$, $\nu$ = 0.4
Xin-Feng et al. (2020)	Friction coefficient of 0.1	Linear elastic: $E = 10.4\,\text {MPa}$, $\nu$ = 0.4
Zhou et al. (2020)	Friction coefficient of 0.2	Linear elastic: $E = 50\,\text {MPa}$, $\nu$ = 0.3
Ayturk and Puttlitz (2011); Du et al. (2016a)	Frictionless	Neo Hooke
Holzapfel and Stadler (2006)	Friction coefficient of 0.06	Neo Hooke
Schmidt et al. (2013)	Frictionless	Mooney-Rivlin 1st order
Barthelemy et al. (2016)	Frictionless	Mooney-Rivlin 2nd order
Noailly et al. (2012)		Mooney-Rivlin 2nd order, incompressible
Noailly et al. (2007)		Asymmetric tension/compression, with hypoeleastic cartilage in compression
Hussain et al. (2010)		Poroelastic using: $E = 10.4\,\text {MPa}$, $\nu$ = 0.4

Of studies which included information on the friction model (Table 1, $n = 30/35$ studies with soft contact and Table 2, $n = 31/45$ studies with cartilage), a majority assumed the contact behaviour to be frictionless $(n = 50/61)$, while only eleven included some friction (with a friction coefficient ranging from 0.01 to 0.2). None of the model with friction specified the type of friction law used.

### Cartilage material model

Of studies which incorporate a 3D deformable cartilage layer, only one did not consider the cartilage as a purely elastic material, but used a poroelastic model instead (Hussain et al. 2010). Most other studies used a linear elastic material law with a large variation in Young’s modulus (median 10.4 MPa, range 10 kPa–3.5 GPa) and Poisson’s ratio (median 0.4, range 0.25–0.45). Those using a hyperelastic material law used a Neo-Hooke model or first- or second-order Mooney–Rivlin model; only one giving information on the compressibility of the material used. Finally, only one study (Noailly et al. 2007) used a material law asymmetric in tension and compression, representing the different behaviour of the cartilage in these configurations.

## Validation processes used in facet joints biomechanics

**Table 3:** Contact information of studies which are unclear about the representation of cartilage (empty cells refer to a lack of relevant information)

References	Friction model
Chen et al. (2015)	Friction coefficient of 0.1
Choi et al. (2016)	Frictionless
Choi et al. (2017)	
Chuang et al. (2012)	Frictionless, initial facet space 0.5 mm
Guo and Li (2019)	Frictionless
Kong et al. (1996)	
Kosalishkwaran et al. (2019)	Frictionless
Li et al. (2020)	
Lin et al. (2014)	Friction coefficient of 0.1
Yang and King (1984)	
Yu et al. (2020)	Friction coefficient of 0.1
Yuchi et al. (2019)	Friction coefficient of 0.01, initial facet space 0.5 mm
Wu et al. (2017)	Frictionless
Zhu et al. (2020)	Friction coefficient of 0.1
Zhang et al. (2018)	Frictionless
Zhu et al. (2015)	

There were as many recent studies ($n = 9/63$ since 2015) as older ones ($n = 9/47$ before 2015) which included some validation of the facet joints biomechanics (Table 4). While ten studies assessed their outcomes with respect to ranges of experimental values available in the literature, 11 assessed their outcomes with respect to FE models outputs, three of which also comparing to experimental data from the literature.

**Table 4:** Study aim and validation work performed for the 18 studies mentioning some validation of facet joints biomechanics (references denoted with * are included in the comparison work in Dreischarf et al. 2014)

References	Study aim	Validation work for facet joints
**Comparison of facet joint force or pressure**		
Azari et al. (2018)	Estimate internal stresses and strains under realistic load conditions	Pooled range of values from eight FE models (Dreischarf et al. 2014)
Ayturk and Puttlitz (2011)*	Validation work	Experimental data (Wilson et al. 2006; Niosi et al. 2008; Sawa and Crawford 2008)
Barthelemy et al. (2016)	Validation of composition-based disc model	Experimental data (Wilson et al. 2006; Niosi et al. 2008; Zhu et al. 2008); computational data (Noailly et al. 2012)
Campbell et al. (2016)	Development of statistical shape modelling	Experimental data (Wilson et al. 2006; Niosi et al. 2008; Sawa and Crawford 2008)
Chen et al. (2009)	Comparison between TDR and fusion	FE models (Chen et al. 2002; Shirazi-Adl 1994)
Goel et al. (1988)	Effect of fixation device	FE models (Shirazi-Adl and Drouin 1987; Yang and King 1984)
Guo et al. (2007)	Effect of denucleation with vibration	FE model (Shirazi-Adl and Drouin 1987)
Guo and Li (2020)	Validation in static and dynamic conditions	Experimental data (Wilson et al. 2006; Niosi et al. 2008; Sawa and Crawford 2008)
Kim et al. (2013)	Effect of facet joints orientation and facet tropism	Experimental data (Wilson et al. 2006)
Khoddam-Khorasani et al. (2018)	Coupling passive FE and active MSK	Pooled range of values from eight FE models (Dreischarf et al. 2014)
Liu et al. (2011)*	Effect of stabilisation system	FE models (Shirazi-Adl et al. 1986; Chen et al. 2009)
Mills and Sarigul-Klijn (2019)	Validation work	Pooled range of values from eight FE models (Dreischarf et al. 2014) and experimental data (Wilson et al. 2006)
Mustafy et al. (2014)	Effect of impact loading rates on load sharing	Experimental data (Jaumard et al. 2011a)
Naserkhaki et al. (2016)	Assessment load share in flexion-extension	Pooled range of values from eight FE models (Dreischarf et al. 2014) and experimental data (Wilson et al. 2006)
Nikkhoo et al. (2020)	Effect of lordosis on fusion	Pooled range of values from eight FE models (Dreischarf et al. 2014)
Wang et al. (1997)	Validation of a viscoelastic model	FE model (Shirazi-Adl and Drouin 1987)
Xu et al. (2017)	Validation work from multiple subjects	Experimental data (Wilson et al. 2006; Niosi et al. 2008; Sawa and Crawford 2008)
**Other comparison**		
Holzapfel and Stadler (2006)	Role of facet curvature	Qualitative comparison of “waviness” of contact pattern

The assessment of the effect of using diverse contact algorithm on facet joints biomechanics was studied by evaluating the contact pattern (Holzapfel and Stadler 2006). Of these 18 studies with validation, none also reported sensitivity analysis on facet joints inputs to outputs of interest. Only one study, whose study aim was to assess the validity of a statistical shape model, reported sensitivity to the geometry of the spinal level of interest (Campbell et al. 2016).

### Comparison with literature experimental data

The source of experimental data from the literature used in validation work was limited, with four experimental studies used for the lumbar spine (Wilson et al. 2006; Niosi et al. 2008; Zhu et al. 2008 from the University of British Columbia, and Sawa and Crawford 2008 from St. Joseph’s Hospital and Medical Center) and one for the cervical spine (Jaumard et al. 2011a).

When comparing outcome of FE models with otherwise published experimental data, all lumbar spine studies used one source of data (Wilson et al. 2006), often alongside others, which includes in its discussion comments about experimental accuracy, reporting a likely overestimation of facet joints forces. This aspect was not acknowledged in validation studies which rarely consider experimental error as a source of error on the validation of computational models. Moreover, for L3/L4, some studies compared their results to both Wilson et al. (2006) and Sawa and Crawford (2008) for which the latter has a mean value almost half of the former. In that case, computational results are usually closer to the highest values (Wilson et al. 2006) than the lower ones (Sawa and Crawford 2008), while the corresponding L1/L2 is validated against Sawa and Crawford (2008)’s data.

Most FE studies compared their outputs with literature reporting data for the same spinal levels (L1–2 or L3–4), however, some reported validation while mixing experimental data from several levels (e.g. Mustafy et al. 2014 comparing one cervical level with the range reported for C2–C6), artificially increasing the range of validity.

### Comparison with literature computational data

Eleven studies provided validation of facet joints outputs against computational data. All validation studies against computational data were able to replicate exactly boundary and loading conditions with respect to study providing target values but none of the target computational studies was specifically validated for facet joints biomechanics.

Five out of six studies performed after 2014 compared their results to the pooled outcomes resulting from the comparison study of eight lumbar models (Dreischarf et al. 2014). This type of validation protocol uses an artificially large variance in the target values for validation. In particular, the range of mean facet joint force values across all eight models in extension and in axial rotation ($\sim 10$ N to $\sim 110$ N and $\sim 40$ N to $\sim 135$  N) was up to twice larger than corresponding experimental data ($\sim 10$ N to $\sim 55$ N and $\sim 55$ N to $\sim 115$ N, Wilson et al. 2006), defining an “easier” validation target.

For five out of six studies performed before 2014, reference studies used were early computational studies (Shirazi-Adl et al. 1986; Shirazi-Adl and Drouin 1987; Yang and King 1984) for which the accuracy of the geometry could be questioned with respect to accuracy of contact forces.

## Discussion

This review of 110 papers from the last 30+ years has shown that there is, to date, no finite element model of the human functional spinal unit (two or more levels) which provides direct validation of the facet joint behaviour. Direct validation is here defined as the direct comparison between a computational result and an experimental result of the same specimen, when the computational model and the experiment match as closely as possible (Jones and Wilcox 2008; Mengoni et al. 2017). All quantitative validation processes for the facet joints in the reviewed studies use comparison with the existing literature data, either computational data or experimental data, all assuming healthy facet joint properties. This process of indirect validation is useful to verify that model outputs are within a range of plausible values. Even when those models are built from patient-specific geometry, it does not demonstrate that the model outputs are valid for a specific geometry, or that the method is able to appropriately capture variation within the population.

One in three studies without facet joint validation focus on the effect of different constructs or disc degeneration on the facet joints. Moreover, while a clinical correlation has been established between intervertebral disc degeneration and facet joints degeneration (Jaumard et al. 2011b; Gellhorn et al. 2013), studies modelling degenerated disc and also including degeneration of the facet joint cartilage material properties or friction behaviour are only those which perform statistical sweeps. As such, most studies evaluate the effect of a disruption for which there is no initial indication of baseline validity or without including concomitant factors. While some qualitative comparison may still be appropriate, there is no indication that quantitative assessment should be taken for granted.

There is often a lack of critical analysis about the methodology used. For example, the facet joint being a synovial joint, it has almost perfect lubrication (Guilak 2005). As such, when healthy, it is likely that the friction coefficient is well below 0.05. Using a higher friction coefficient may be the result of needing to reduce sliding for computational stability rather than a representation of a physical characteristic. When such parameters choices do not seem to be based on the physics of the problem, more should be done in discussing “the art of modelling” and modelling assumptions openly (i.e. choices made so that a model solution can converge). This would contribute to a more honest discussion on the capacity of the modelling approach chosen.

Less than one in five studies provide sufficient details on the facet joint behaviour modelling assumptions (material parameters or pressure-overclosure model and computational representation of friction). This lack of information is somewhat contributing to reduced confidence in the outcomes and mostly to poor reproducibility. The lack of information on soft contact models is the most common, often only mentioning the use of “soft contact” or “nonlinear contact”. This may be due to using default approaches in commercial finite element software without a clear understanding of what these are or of what they represent physically. The use of software-specific terminology reinforces that interpretation. Issues linked with poor reproducibility in joint biomechanics is not specific to modelling of the facet joints. For example, the likely shortcomings of natural knee modelling are the basis of a large multi-centre reproducibility study, assessing the effect of the “art of modelling” on model outputs variability (Erdemir et al. 2019).

There is often a trade-off between the accuracy of cartilage geometry and materials in facet joint modelling assumptions. Including a complex representation of the cartilage, combined with a nonlinear material law may increase the overall non-linearity of a computational model to a point where solving becomes too difficult. As such, models which represent the cartilage geometry explicitly often have linear material properties, while models with nonlinear soft contact assumptions do not include any inhomogeneity in the cartilage anatomy (in particular its thickness). The cartilage geometry, even when explicitly included, is always an approximation, based on general anatomical knowledge such as setting a given initial joint space or an average cartilage thickness. Sensitivity studies including heterogeneous thickness of the cartilage (Woldtvedt et al. 2011; Niemeyer et al. 2012) showed that while the ranges of motion were not altered with respect to homogeneous cartilage, outputs related to contact and load share were highly affected. Variable cartilage thickness models could be done by incorporating data from MR images to models that are often built from CT images. This comes with the difficulty of poor anatomical resolution in clinical MR (where standard MR protocols do not capture many slices within each facet joints) and may therefore be more suited for cadaveric studies where high-resolution MR can be acquired with very few artefacts.

There is no extensive sensitivity study on the effect that cartilage material law has on facet outputs of interest. As the initial facet joint space and the cartilage thickness seem to have a major effect on contact pressure obtained during normal range of movements (Niemeyer et al. 2012), the effect of material law may be secondary. In theory, material models for the cartilage should represent its different behaviour in tension and compression (Noailly et al. 2007). Using a soft-contact model for the cartilage has the benefit to include this aspect by default, while models with an explicit representation of the cartilage should include this behaviour in the material law. This effect needs to be included if the cartilage may sustain loads which are not compressive. However, as most contact models used are frictionless and inactive once the surfaces are not overlapping, it is unlikely that the facet cartilage sustain loads other than compressive loads. As such, including the different behaviour in tension and compression may only have a secondary effect.

In a validation process comparing data to the literature, the aim is to obtain model outputs within a range of equivalent literature values. By nature of working with natural tissues and somewhat different testing protocols, there is a large variability in experimental data on facet joints found in the literature. Comparing computational outcome to experimental data obtained with a protocol as similar as the computational model is therefore critical when choosing what experimental data to use in validation studies. Data to compare against should not be chosen a-posteriori to fit computational outcomes. Computational studies are often unable to replicate facet joint forces of two adjacent levels from a unique data source. This lack of consistency between levels does not provide confidence that one model can replicate one experimental protocol but rather that models are able to replicate average values coming from different testing protocols. There is also a large variability in computational data on facet joints in the literature, which increases by including studies which are not specifically validated against facet joint outputs. Performing validation studies against computational data which is not known to be valid for facet joint outputs increases the risk of a model being wrongly deemed valid and should be avoided.

Validation can be done comparing model outcomes to equivalent data from the literature or to data corresponding directly to the specimen being modelled. While most validation studies analysed here replicate as closely as possible an equivalent experimental or computational protocol, they do not report on how sensitive the model outputs are to its inputs or which assumptions can be modified and still produce a valid outcome. When building model geometries from patient- or specimen-specific images, this means that there is no indication on the patient-specificity of the outcomes produced by a modelling method deemed to be valid with respect to literature data. A validation process comparing a model to its direct experimental equivalent has the benefit that the specificity of the validation can be assessed (Jones and Wilcox 2008). When performing such a direct validation across several specimens, it also has the advantage that it provides confidence in the ability to model the specimen-to-specimen variation in the outputs of interest (Mengoni 2020b). However, there is often a trade-off between having a model representative of a realistic, often uncertain, situation and replicating closely a given specimen and its testing conditions (Cooper et al. 2019).

While model validation is always limited to a given set of inputs and outputs, using models or methodologies outside their validation range is where computational modelling can become useful, providing new information. This should be performed with clear identification of the context of use (Viceconti et al. 2020), justifying using appropriate variation in inputs or outputs with respect to the validation study. Systematically providing comprehensive information on model methodologies (“the art of modelling”) and on the physical data used in validation studies (input and output data) would provide better confidence in the context of use of “valid” models and increase the possibility to directly compare model methodologies against the same physical data. In the case of facet joints biomechanics for which no direct validation study exists, systematically sharing data, including 3D imaging, through open-repositories would allow more users to demonstrate that their model outputs are valid for specific inputs, and that their method is able to appropriately capture variation within the population.

## Data Availability

All data presented in this paper, as well as the equivalent data for the 52 studies which did not contain outputs of interest for the facet joints, are available at the University of Leeds repository (Mengoni 2020a).
